# Anxiety and Worries of Individuals with Down Syndrome During the COVID-19 Pandemic: A Comparative Study in the UK

**DOI:** 10.1007/s10803-022-05450-0

**Published:** 2022-02-01

**Authors:** V. Sideropoulos, H. Kye, D. Dukes, A. C. Samson, O. Palikara, J. Van Herwegen

**Affiliations:** 1 Department of Psychology and Human Development, UCL, Institute of Education, University College London, London, UK; 2 Institute of Special Education, University of Fribourg, Fribourg, Switzerland; 3 Swiss Center for Affective Sciences, University of Geneva, Geneva, Switzerland; 4 Faculty of Psychology, Unidistance Suisse, Brig, Switzerland; 5 Department for Education Studies, University of Warwick, Coventry, UK

**Keywords:** COVID-19, Down syndrome, Anxiety, Worries, SEND, Predictors

## Abstract

The present study explored the effects of the pandemic on individuals with Down Syndrome (DS; n = 67) compared to other groups with Special Education Needs and Disabilities (SEND; n = 48) and their Typically Developing Siblings (TDS; n = 56). In total, 115 caregivers reported on their own anxiety and worries and of their children. Anxiety levels for individuals with DS appeared to be lower compared to other SEND populations and to TDS. In terms of worries, individuals with DS worried more about social-related worries but worried less about family-related aspects compared to the other groups. In sum, individuals with DS might show less anxiety but still worried more about specific aspects related to the impact of COVID-19 pandemic on their lives.

**Supplementary Information:**

The online version contains supplementary material available at 10.1007/s10803-022-05450-0.

## Introduction

Around the world, there has been much research on mental health during the COVID-19 pandemic and the World Health Organisation (WHO) has highlighted the increased rates for symptoms of anxiety (6–51%) and depression (15–48%) (WHO, 2021). According to previous research, early life adversities are a key factor for either short- or long-term mental health difficulties (Scott, 2011). During the COVID-19 pandemic, some of the most common adversities that are experienced by the majority of the population include unemployment and financial instability; missed education and lost prospects; social isolation and fear of life-threatening disease in self or loved ones (WHO, 2021). In fact, the impact of the COVID-19 pandemic varies across the globe, depending on the population. For instance, evidence suggests that those with pre-existing mental health problems and other behavioural disorders who experienced adversities such as financial instability and/or missed education were at a higher risk of mental health problems during the COVID-19 pandemic (Neelam et al., 2021; WHO 2021). 

However, there has been limited research on how the COVID-19 pandemic affects individuals with neurodevelopmental disorders. Reports suggests that families of individuals with Special Education Needs and Disabilities (SEND) have been greatly impacted by the pandemic as there was a change in routine and a lack of access to support networks as well as added caring responsibilities which caregivers found challenging to combine with other duties (e.g., working from home) (Asbury et al., 2021). There is also evidence that suggests increased rates of anxiety and elevated worries for individuals with SEND after the first wave of COVID-19 in China (Su et al., 2021). Sideropoulos et al. (2021) highlighted the increased levels of anxiety in individuals with SEND during the first months of the pandemic in the UK. In addition, elevated worries about school closures and loss of institutional support for individuals with SEND during the lockdowns have been also discussed in the literature (Sideropoulos et al., 2021, Su et al., 2021).

Whist mathematical models of epidemics show the effect of multiple lockdowns can be effective (Scala, 2021), mental health researchers raised concerns around their impact on mental health as many countries, including the UK, were undergoing a series of lockdowns (Adams-Prassl et al., 2020; Rossi et al., 2020; Thakur et al., 2020). The effects of confinement and severe physical or social restrictions have shown deleterious effects on mental health. Such research spans situations such as solitary confinement (Chadick et al., 2018; Reiter et al., 2020), segregation (Rubio et al., 2021; Valentine et al., 2019), and polar expeditions (Palinkas & Suedfeld, 2008; Rubio et al., 2021). Though the impact of the COVID-19 pandemic and its restrictions have caused significant elevated rates of mental health to individuals with SEND (Panchal et al., 2021), to our knowledge there is no research on the effects of the pandemic on individuals with Down Syndrome and the challenges and difficulties families of individuals with Down Syndrome have experienced during the COVID-19 pandemic.

Down syndrome (DS) is a genetic disorder caused by an additional chromosome 21 occurring spontaneously for approximately 1 in 1000 live births, resulting in intellectual disability. There are large individual differences in DS in general intellectual ability. Around 80% of individuals with DS have moderate intellectual disability, but some fall within the severely impaired range and others overlap with typical development (Chapman & Hesketh, 2000; Di Nuovo & Buono, 2009; Pueschel, 1995; Thomas et al., 2020; Zampini & D’Odorico, 2013). DS is typically characterized by particular difficulty with expressive language and cognitive delay (Chapman, 1997; Daunhauer & Fidler, 2011) with these difficulties becoming more pronounced overtime due to a slower pace of development (D’Souza et al., 2020; Dykens et al., 2006; Fidler et al., 2006; Miller, 1999; Oliver & Buckley, 1994; Startin et al., 2020). Individuals with DS often show poor short-term memory but relative strengths in relation to visual -spatial difficulties, sensory processing, behavior and social responsiveness (Foley et al., 2016; Tassé et al., 2016). There is wide variability in the language abilities of those with DS from being non-verbal to developing relatively large vocabularies. In addition, individuals with DS are reported to exhibit fine and gross motor problems or delays in adaptive behaviors; such as feeding and dressing oneself (Marchal et al., 2016).

Scholars have extensively discussed the emotional and behavioral challenges children with DS experience (Gameren-Oosterom et al., 2011; Naerland et al., 2016). It is common for children with DS to have difficulties with verbal working memory, speaking, writing and arithmetic (Cuskelly et al., 2017; Faragher, 2017; Næss et al., 2011; Rice et al., 2005; Will et al., 2019). Other studies also report problems with social withdrawal, social skills and disobedient behavior (Barisnikov & Lejeune, 2018; Coe et al., 1999; Galeote et al., 2011). Despite those challenges, children with DS score significantly lower on anxiety and depression when compared to typically developing peers (Gameren-Oosterom et al., 2011). However, this could be a result of numerous factors ranging from other concomitant health problems to access to care and more, which are yet to be explored in the DS literature.

Recent research which draws on data from the COVID-19 pandemic in Italy suggests that individuals with DS experienced increased rates of depressive symptoms and social withdrawal than before the first national lockdown (Villani et al., 2020). It should be noted that several confounding factors may contribute to either the positive or negative elevated levels in individuals with DS (Esbensen & Seltzer, 2011), including age and sex, which have not yet been researched (Sáncbez-Teruel & Robles-Bello, 2020). Hence, there is a vital need to develop a better understanding of the factors associated with syndrome-specific impacts on the mental health of those individuals. For example, at a functional level, scores on the Instrumental Activities of Daily Living scale (IADLs) worsened since the beginning of the pandemic for individuals with DS, whilst scores on the Activities of Daily Living scale (ADLs) did not change significantly. Whilst ADLs include basic self-care tasks such as bathing, IADLs include more complex tasks related to one’s ability to live independently (e.g., shopping, using public transportation; Villani et al., 2020). Nevertheless, the research is primarily focused on how these factors contribute to the caregivers’ mental health rather than on the impact of those factors on the individuals with DS (Esbensen & Seltzer, 2011).

Regardless of the limited evidence available relating to the impact of COVID-19 on individuals with DS, research has previously demonstrated the uniqueness of caring for an individual with DS. For example, Fidler et al. (2000) looked at three different groups of children with Intellectual Disability and their families. They compared the scores of maladaptive behavior in children with DS, Williams Syndrome (WS) and Smith-Magenis syndrome, as well as parental outcomes such as Parent and Family Problems and Parental Pessimism. Children with DS exhibited significantly lower rates of maladaptive behavior than the other two groups. Moreover, among the families participating in the study, the families of children with DS scored the lowest in overall pessimism, and in parent and family problems. The study also found that predictors of stress varied among the three groups, where only age significantly predicted family stress in families of individuals with DS, whilst for families of individuals with Smith-Magenis syndrome, maladaptive behavior predicted stress levels, while in families of individuals with WS, both factors predicted stress levels. The authors suggested that this difference reflects the lower levels of maladaptive behavior found in children with DS. Other studies echo the finding that children with DS not only exhibit lower psychiatric levels of mental health (Spendelow, 2011) but also that they demonstrate the uniqueness of children with DS (Corrice & Glidden, 2009).

A different way to address the question of what accounts for the different levels of psychosocial wellbeing observed in individuals with DS is the investigation of caregivers’ wellbeing. Research has demonstrated the links between caregivers’ mental health and the way they perceive their children’s wellbeing (Neece et al., 2012) as well as the different factors that contribute to parental stress depending on the neurodevelopmental condition their child has (Ashworth et al., 2019). Caregivers of individuals with DS often report experiencing lower levels of stress (e.g. Esbensen & Seltzer, 2011; Kasari & Sigman, 1997), having less pessimistic outlooks regarding their child’s future (Fidler et al., 2000), perceiving less temperamental difficulties in their children (Kasari & Sigman, 1997), and having greater and more satisfying social networks and support (Hauser-Cram et al., 2001), where these differences were reported in relation to caregivers of children with other developmental conditions, or typically developing children. This effect was named the Down Syndrome Advantage. Whilst exceptions have been found to this phenomenon in the literature (Cunningham, 1996; Esbensen et al., 2008; Gath, 1990; Greenberg et al., 2004; Roach et al., 1999; Sanders & Morgan, 1997), research findings tend to report more positive and less negative wellbeing outcomes for caregivers of individuals with DS compared to mothers of children with other developmental and intellectual disabilities (Esbensen & Seltzer, 2011). In addition, typically developing siblings (TDS) of individuals with DS report more positive wellbeing outcomes than TDS of individuals with autism, in terms of their depressive symptoms, warmth within the sibling relationship, and higher levels of positive affect towards their sibling (Hodapp & Urbano, 2007; Orsmond & Seltzer, 2007).

In sum, whilst previous research has highlighted elevated levels of anxiety in the SEND population during the COVID-19 pandemic (Asbury et al., 2021; Sideropoulos et al., 2021), there is a lack of research on how the experiences of those with DS compare to other SEND groups. Given that previous studies have shown that individuals with DS may experience and respond to averse situations differently than other groups of SEND, it is not clear whether this also holds for how they experience the impact of COVID19. It is therefore important to investigate the mental health, specifically anxiety, of individuals with DS during the COVID-19 pandemic, in order to make comparisons to other groups of individuals with SEND.

## The present study

The primary aim of the present study was to explore the impact of the COVID-19 pandemic on the anxiety levels of individuals with DS and their families using cross-sectional data from the 3rd national lockdown in the UK (early January 2021 to May 2021; COVID-19 Response—Spring 2021 (Summary), 2021). We hypothesized that those with a diagnosis of DS would report lower levels of anxiety during the third lockdown compared to individuals with a different SEND diagnosis. Due to the lack of literature in the area, we also compared individuals with DS to their TDS in terms of anxiety. We hypothesized that individuals with DS would exhibit similar levels of anxiety to their TDS. Furthermore, we investigated the predictors for anxiety. Based on previous research on the impact of COVID-19 on individuals with SEND (Sideropoulos et al., 2021), we hypothesized that those with an existing anxiety disorder, who were aware of COVID-19 and who had an anxious caregiver would report higher levels of anxiety across all groups (Sideropoulos et al., 2021). Finally, we expected that the individuals with DS would score lower across the many and varied worries we measured, compared to the individuals with other SEND as well as their TDS.

## Methods

### Participants

115 caregivers (97.53% female) of 171 young individuals (115 children with SEND of which 56 had a TDS) completed an online survey. The caregivers were aged 23 to 66 (M = 46.78, SD = 7.96) and 33.91% (n = 39) were educated to a university degree level (e.g., having completed a Bachelors degree). There was no statistically significant association between caregiver’s educational qualification and the three groups we used in our analysis: *χ*(5) = 4.46, *p* = 0.48.

The caregivers were recruited through various means of communication such as social networks, social media, word-of-mouth, by emails to special education institutions as well as through support groups such as Williams Syndrome Foundation, Down Syndrome Association UK, and ADHD Foundation UK.

When looking at the total SEND population, parents reported that 21.74% (n = 25, 6 of those were individuals with DS) of their children had previously received a diagnosis of anxiety and 70.43% (n = 81) were aware of the COVID-19 pandemic. It is important to note that the individuals with SEND may not have consciously understood any changes to their routine caused by the COVID-19 pandemic. Yet, they still may have experienced higher anxiety as a result of the new routines caused by the pandemic (e.g., wearing masks, frequent testing) and thus they were included in the analyses.

All the caregivers reported that their child with SEND had received a formal diagnosis (reported in Table 1). Individuals with SEND (32.65%, n = 16 female) ranged in age from 2 to 25 years old (M = 13.37, SD = 6.48). As can be seen from Table 1, 58.3% (n = 67) had a diagnosis of DS (47.83%, n = 33 female). Due to the hypotheses and aims of this paper, we grouped all the other diagnoses (41.74%, n = 48) into a group (named “other SEND”).

**Table 1:** Overview of diagnosis of children with Special Education Needs and Disabilities (SEND)

Type of diagnosis	Frequency	Percent	Valid percent	Cumulative percent
Autism spectrum disorder	8	7.0	7.0	7.0
Down syndrome	67	58.3	58.3	65.2
Intellectual disability (not otherwise specified)	3	2.6	2.6	67.8
Williams syndrome	15	13.0	13.0	80.9
Attention-deficit disorder (with or w/out hyperactivity)	4	3.5	3.5	84.3
Other syndrome/diagnosis:	18	15.7	15.7	100.00
Total	115	100.00		

Out of the 115 caregivers, 56 also completed the survey for a TDS (63.16%, n = 36 female) in the family. The TDS had a similar age range as the total SEND population; 3–24 years (M = 13.11, SD = 5.88). Only 5.45% (n = 6) of the TDS were diagnosed with an anxiety disorder and all of them were aware of COVID-19 (n = 54 with two missing data cases).

We also measured caregivers’ anxiety on a 5-Likert scale with higher scores denoting higher levels of anxiety. Although caregivers of individuals with DS (M = 3.75, SD = 1.10) reported higher levels of anxiety compared to caregivers of individuals with other SEND (M = 3.42, SD = 1.37) and caregivers who also reported that they have a typically developing child (M = 3.58, SD = 1.25), these differences were not significant, *F*(2,227) = 1.07, *p* = 0.34.

### Materials and Procedure

Caregivers completed an anonymous cross-sectional survey (similar to Sideropoulos et al., 2021) through Qualtrics between 29th of January 2021 and 29th March 2021 which coincided with the 3rd national lockdown in the UK. Their participation was entirely voluntary as well as anonymous.

This survey contained a range of open-ended and closed questions over four key sections of which only three were used for this study: (a) demographic questions about the children; (b) COVID-19 related questions (not used for this study); (c) concerns and worries of the participating caregiver and d) of their children.

The thirteen questions around worries were informed by the wellbeing categories as defined by Schalock (1996) and included worries related to social inclusion (e.g., not being able to meet others), physical wellbeing (e.g., worries about catching COVID-19 and own health), interpersonal relations (e.g., worry about family conflict and others becoming ill), material wellbeing (e.g., financial worries), emotional wellbeing (e.g., worries about boredom), self-determination (e.g., loss of routine), and personal development (e.g., loss of institutional support). These were grouped into the following categories: Health Related Worries, Social Related Worries, School Closure Related Worries and Family Related Worries. All worries were rated on a scale from 1 to 5 (with 1; “not concerned at all”, to 5; “very concerned”).

Participants were asked to rate their own and their children’s anxiety and worries over three time-points: (a) before March 2020 (pre-pandemic); (b) during March 2020 (initial lockdown and start of the pandemic) and (c) now (January 2021 to March 2021).

All the materials can be accessed on the Open Science Framework website (Van Herwegen et al., 2020a, 2020b): https://osf.io/5nkq9/.

### Ethics

Ethical approval for the study was obtained from Ethics Commission of UniDistance, Switzerland before the start of the study. Respondents provided online consent to take part in the online study and they were free to withdraw at any stage.

## Results

### Effect of Time on Anxiety for Individuals with DS, Other SEND and TDS

A mixed-model ANOVA was computed to determine the effect of time on anxiety for our three groups (participants with DS, those with a different SEND and their TDS). There was a significant effect for time in our model which indicates that there was a difference in the reported anxiety levels for our groups over time. Mauchly’s Test of Sphericity indicated that the assumption of sphericity had been violated: χ^2^ (2) = 18.46, *p* < 0.001. Hence, the degrees of freedom had to be adjusted using the Huynh–Feldt correction; (ε = 0.91); *F*(1.83, 30,136) = 55.73,* p* < 0.001, η^2^ = 0.08.

There was also a main effect for Group, *F*(2,165) = 10.60, *p* < 0.001, η^2^ = 0.07 indicating that there was a difference between the groups’ reported anxiety. As reported in Table 2, those with DS had lower reported anxiety compared to other individuals with SEND overall (*p* < 0.001). There was no significant difference between the young individuals with DS and the TDS group (*p* > 1.00). In contrast, there was a significant difference between young people with other SEND and the TDS group (*p* < 1.13e−3).

**Table 2:** Post Hoc Comparisons for Time, Group (Typically developing siblings = TDS, Down syndrome = DS, Other SEND) and Group * Time

				95% CI for mean difference						
			Mean difference	Lower	Upper	SE	t	Cohen's d	*p*_bonf_	
Time	Before March 2020	During March 2020	− 0.82	− 1.03	− 0.60	0.09	− 9.09	− 0.70	< .001***	
		Now March 2021	− 0.83	− 1.04	− 0.61	0.09	− 9.19	− 0.71	< .001***	
	During March 2020	Now March 2021	− 9.04e −3	− 0.23	0.21	0.09	− 0.10	− 7.75e −3	1.00	
Group	DS	Other SEND	− 0.84	− 1.31	− 0.38	0.19	− 4.38	− 0.34	< .001***	
		TDS	− 0.11	− 0.56	0.34	0.19	− 0.60	− 0.05	1.00	
	Other SEND	TDS	0.73	0.24	1.22	0.20	3.63	0.28	1.13e−3**	
Group * Time	DS, before March 2020	Other SEND, before March 2020	− 0.90	− 1.65	− 0.16	0.23	− 3.92		3.89e−3**	
		TDS, Before March 2020	0.22	− 0.49	0.94	0.22	1.00		1.00	
		DS, during March 2020	− 0.61	− 1.07	− 0.16	0.14	− 4.34		< .001***	
		Other SEND, during March 2020	− 1.54	− 2.29	− 0.80	0.23	− 6.69		< .001***	
		TDS, during March 2020	− 0.98	− 1.70	− 0.27	0.22	− 4.43		< .001***	
		DS, now March 2021	− 0.76	− 1.22	− 0.31	0.14	− 5.40		< .001***	
		Other SEND, now March 2021	− 1.46	− 2.20	− 0.71	0.23	− 6.32		< .001***	
		TDS, now March 2021	− 0.94	− 1.66	− 0.23	0.22	− 4.26		< .001***	
	Other SEND, before March 2020	TDS, before March 2020	1.13	0.35	1.91	0.24	4.66		< .001***	
		DS, during March 2020	0.29	− 0.45	1.04	0.23	1.27		1.00	
		Other SEND, during March 2020	− 0.64	− 1.18	− 0.10	0.17	− 3.79		6.38e−3**	
		TDS, during March 2020	− 0.08	− 0.86	0.70	0.24	− 0.32		1.00	
		DS now March 2021	0.14	− 0.60	0.89	0.23	0.62		1.00	
		Other SEND, now March 2021	− 0.55	− 1.10	− 0.01	0.17	− 3.29		0.04*	
		TDS, now March 2021	− 0.04	− 0.82	0.74	0.24	− 0.17		1.00	
	TDS, before March 2020	DS, during March 2020	− 0.83	− 1.55	− 0.12	0.22	− 3.76		7.28e−3**	
		Other SEND, during March 2020	− 1.76	− 2.54	− 0.98	0.24	− 7.30		< .001***	
		TDS, during March 2020	− 1.20	− 1.71	− 0.70	0.16	− 7.67		< .001***	
		DS, now March 2021	− 0.98	− 1.70	− 0.27	0.22	− 4.43		< .001***	
		Other SEND, now March 2021	− 1.68	− 2.46	− 0.90	0.24	− 6.95		< .001***	
		TDS, now March 2021	− 1.17	− 1.67	− 0.66	0.16	− 7.43		< .001***	
	DS, during March 2020	Other SEND during March 2020	− 0.93	− 1.67	− 0.19	0.23	− 4.04		2.46e−3**	
		TDS, during March 2020	− 0.37	− 1.09	0.34	0.22	− 1.67		1.00	
		DS, now March 2021	− 0.15	− 0.60	0.31	0.14	− 1.06		1.00	
		Other SEND, now March 2021	− 0.85	− 1.59	− 0.10	0.23	− 3.67		0.01*	
		TDS, now March 2021	− 0.33	− 1.05	0.38	0.22	− 1.50		1.00	
	Other, SEND, during March 2020	TDS, during March 2020	0.56	− 0.22	1.34	0.24	2.32		0.76	
		DS, now March 2021	0.78	0.04	1.53	0.23	3.39		0.03*	
		Other SEND, Now March 2021	0.09	− 0.46	0.63	0.17	0.51		1.00	
		TDS, now March 2021	0.60	− 0.18	1.38	0.24	2.47		0.50	
	TDS, during March 2020	DS, now March 2021	0.22	− 0.49	0.94	0.22	1.00		1.00	
		Other, SEND. now March 2021	− 0.48	− 1.26	0.30	0.24	− 1.97		1.00	
		TDS, now March 2021	0.04	− 0.47	0.54	0.16	0.24		1.00	
	DS now March 2021	Other SEND, Now March 2021	− 0.70	− 1.44	0.05	0.23	− 3.02		0.10	
		TDS now March 2021	− 0.18	− 0.90	0.53	0.22	− 0.83		1.00	
	Other, SEND, now March 2021	TDS, now March 2021	0.51	− 0.27	1.29	0.24	2.12		1.00	

*p* value and confidence intervals adjusted for comparing a family of 36 estimates (confidence intervals corrected using the Bonferroni method)

Cohen’s d does not correct for multiple comparisons

*p < .05, **p < .01, ***p < .001

**Fig. 1:**
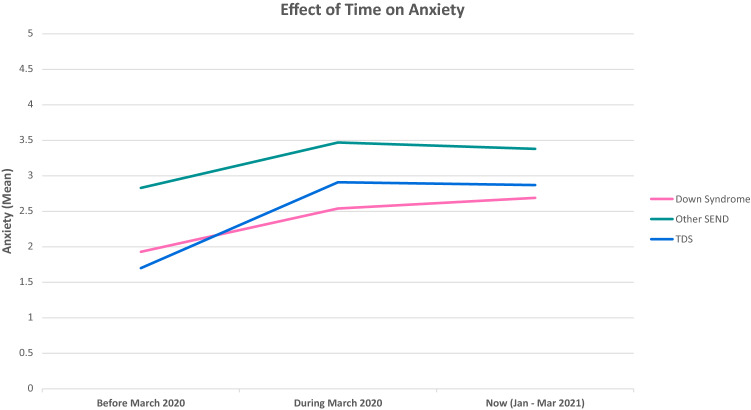
Visualisation of the effect of time on reported child anxiety

In addition, our mixed model ANOVA indicated that there was also a significant Time X Group interaction. Mauchly’s Test of Sphericity indicated that the assumption of sphericity had been violated: χ^2^ (2) = 18.46, *p* < 0.001. Hence, the degrees of freedom were adjusted using the Huynh–Feldt correction; (ε = 0.91); *F*(3.65,301.36) = 3.03, *p* < 0.002, η^2^ = 9.24e−3. Planned post-hoc comparisons for the effect of time, group and the interaction were computed and are reported in Table 2. As can be seen in Fig. 1, individuals with DS scored lower at all time points, when compared with those with a different SEND diagnosis. When comparing the individuals with DS to the TDS group, we can see that again the individuals that score lower are those with DS apart from the first time point (Before March 2020) at which time point TDS scored lower.

In terms of what differences occurred across the timeline of the pandemic, individuals with DS differ significantly from those with other SEND (*p* < 0.3.89e−3) but not the TDS group before the pandemic (*p* > 1.00). At the start of the pandemic (March 2020), there was again a significant difference between individuals with DS and those with other SEND (*p* <  2.46e−3) but not with TDS (*p* > 1.00). However, at the final time point (March 2021), there was no longer a significant difference between those with DS and other SEND (*p* > 0.10) and again no significant difference from the TDS (*p* > 1.00). 

However, in terms of changes over time within the DS group, there was a significant difference between Before March 2020 and During March 2020 (*p* < 0.001), highlighting elevated levels of anxiety during the COVID-19 pandemic, but there was no longer a significant difference between during March 2020 and Now March 2021 (*p* > 1.00). For the young individuals with other SEND, we see a significant difference between Before March 2020 and During March 2020 (*p* < 0.6.38e−3) with scores increasing, but, similarly to the DS group, we see that there was no longer a significant difference between March 2020 and Now March 2021 (*p* > 1.00). Finally, scores increased significantly between Before March 2020 and During March 2020 (*p* < 0.001) for the TD group. Yet, similarly to the other groups there was no significant difference between March 2020 and Now March 2021 (*p* > 1.00).

#### Predictors of Anxiety

Multiple linear regressions were computed for each of our groups to predict anxiety levels during the time the survey was completed (time-point 3) from the following variables: age, gender, health status, awareness of COVID-19, diagnosis of anxiety disorder and the caregiver’s anxiety for the same time-point. To maximise the value of the data we have available, we excluded cases using a pairwise deletion. The reported models can be found in Table 3 and the coefficients from the models in Table 4.

**Table 3:** Multiple linear regression models summary

Models	
1 (DS)	*F*(6,60) = 6.354, *p* < .001, R^2^ = .389 and R^2^ adjusted = .327
2 (Other SEND)	*F*(6,40) = 12.370, *p* < .001, R^2^ = .650 and R^2^ adjusted = .597
3 (TDS)	*F*(5,48) = 4.790, *p* < .001, R^2^ = .333 and R^2^ adjusted = .263

*DS* Down Syndrome, *TDS* Typically Developing Sibling

**Table 4:** Coefficients ^a^ of all the multiple linear regression models

Model		Unstandardized coefficients		Standardized coefficients			Collinearity statistics	
		B	Std. Error	Beta	t	Sig	Tolerance	VIF
1	(Constant)	− .141	1.232		− .114	.909		
	Age	− .017	.023	− .093	− .736	.464	.638	1.567
	Gender	− .086	.276	− .033	− .312	.756	.887	1.128
	Health status	− .346	.172	− .214	− 2.017	.048*	.903	1.107
	Caregiver’s anxiety	.225	.125	.191	1.794	.078	.899	1.113
	Anxiety disorder	1.753	.470	.390	3.726	.000***	.931	1.074
	COVID-19 awareness	1.087	.352	.398	3.090	.003**	.616	1.624
2	(Constant)	1.947	1.148		1.696	.098		
	Age	− .022	.025	− .095	− .917	.364	.811	1.233
	Gender	− .378	.270	− .136	− 1.402	.168	.936	1.068
	Health status	− .467	.138	− .359	− 3.384	.002**	.777	1.287
	Caregiver’s anxiety	.277	.109	.285	2.539	.015*	.697	1.435
	Anxiety disorder	1.039	.301	.388	3.453	.001***	.692	1.445
	COVID-19 awareness	.870	.336	.280	2.589	.013*	.746	1.340
3	(Constant)	.251	1.669		.151	.881		
	Age	− 8.944E−5	.025	.000	− .004	.997	.970	1.031
	Gender	.154	.318	.060	.484	.631	.912	1.096
	Health status	− .060	.290	− .026	− .208	.836	.916	1.091
	Caregiver’s anxiety	.119	.125	.117	.951	.346	.914	1.095
	Anxiety disorder	2.005	.518	.511	3.873	.000***	.798	1.252

^a^Dependent variable: anxiety (Jan–March 2021)

^*^p < .05, **p < .01, ***p < .001

### Linear Regression to Predict Anxiety for Down Syndrome (Model 1)

In the multiple linear regression analysis (Tables 3, 4) for the DS group, we can see an association of health status (b = − 0.346 and β = − 0.214), diagnosis of anxiety disorder (b = 1.753 and β = 0.390) as well as awareness of COVID-19 (b = 1.087 and β = 0.398). However, none of the other factors were associated with anxiety for time-point 3 for the DS group. Individuals with DS who had health problems were more likely to show higher anxiety as were those who were diagnosed with an anxiety disorder and were aware of COVID-19 during the time the survey was completed.

### Linear Regression to Predict Anxiety for Individuals with Other SEND (Model 2)

For the multiple linear regression (Tables 3, 4) for the other SEND group, we see a similar pattern where again health status (b = − 0.467 and β = − 0.359), diagnosis of anxiety disorder (b = 1.039 and β = 0.388) and awareness of COVID-19 (b = 0.870 and β = 0.280) were associated with higher levels of anxiety, but in this model, we also see caregivers’ anxiety for the same time-point to be a significant factor (b = 0.277 and β = 0.285). Hence, individuals in the other SEND group with anxious caregivers and health problems were more likely to score higher on anxiety as well as those who were diagnosed with an anxiety disorder and were aware about COVID-19.

### Linear Regression to Predict Anxiety for Typically Developing Siblings (Model 3)

The final multiple linear regression (Tables 3, 4) for the TDS group indicated that only the diagnosis of anxiety (b = 2.005 and β = 0.511) was associated with elevated levels of anxiety, but none of the other factors. It is important to mention that the variable awareness of COVID-19 was omitted by SPSS v.17 during the computation of the model, due to the many missing observations. Overall, it is clear from the model that TDS with a diagnosis of an anxiety disorder were more likely to exhibit higher levels of anxiety.

#### Reported Worries

**Fig. 2:**
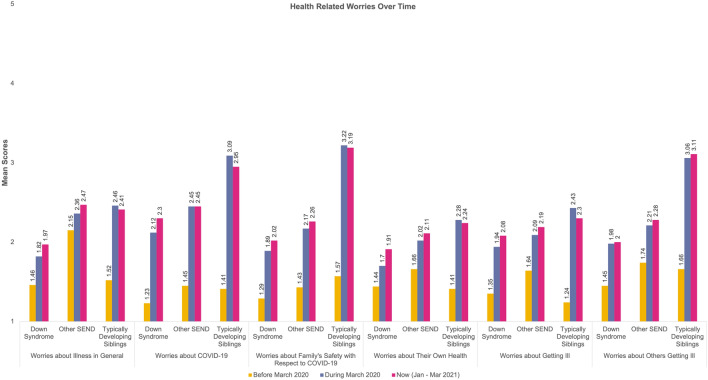
Visualisation of change over time for reported health related worries

**Fig. 3:**
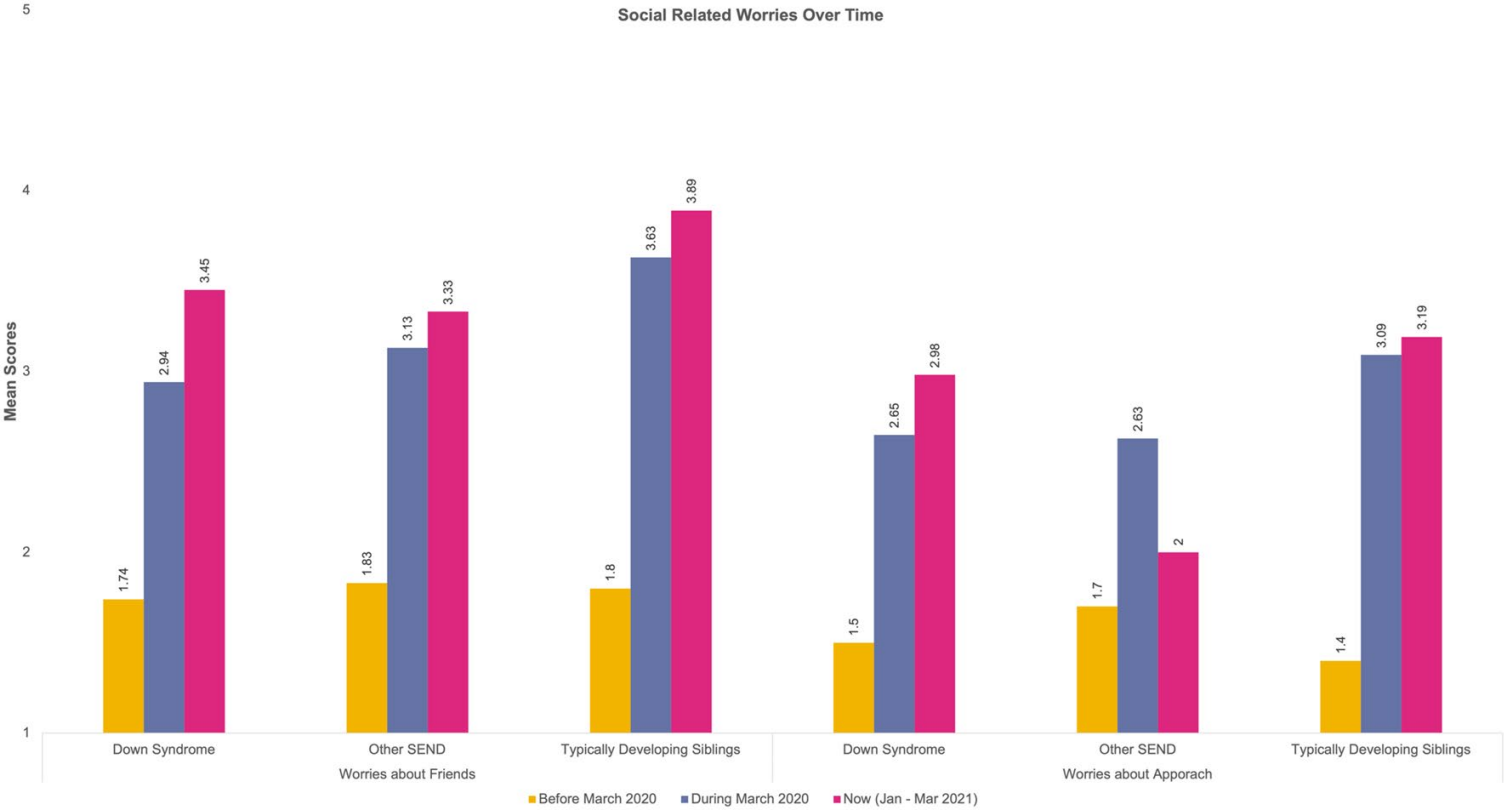
Visualisation of change over time for reported social related worries

**Fig. 4:**
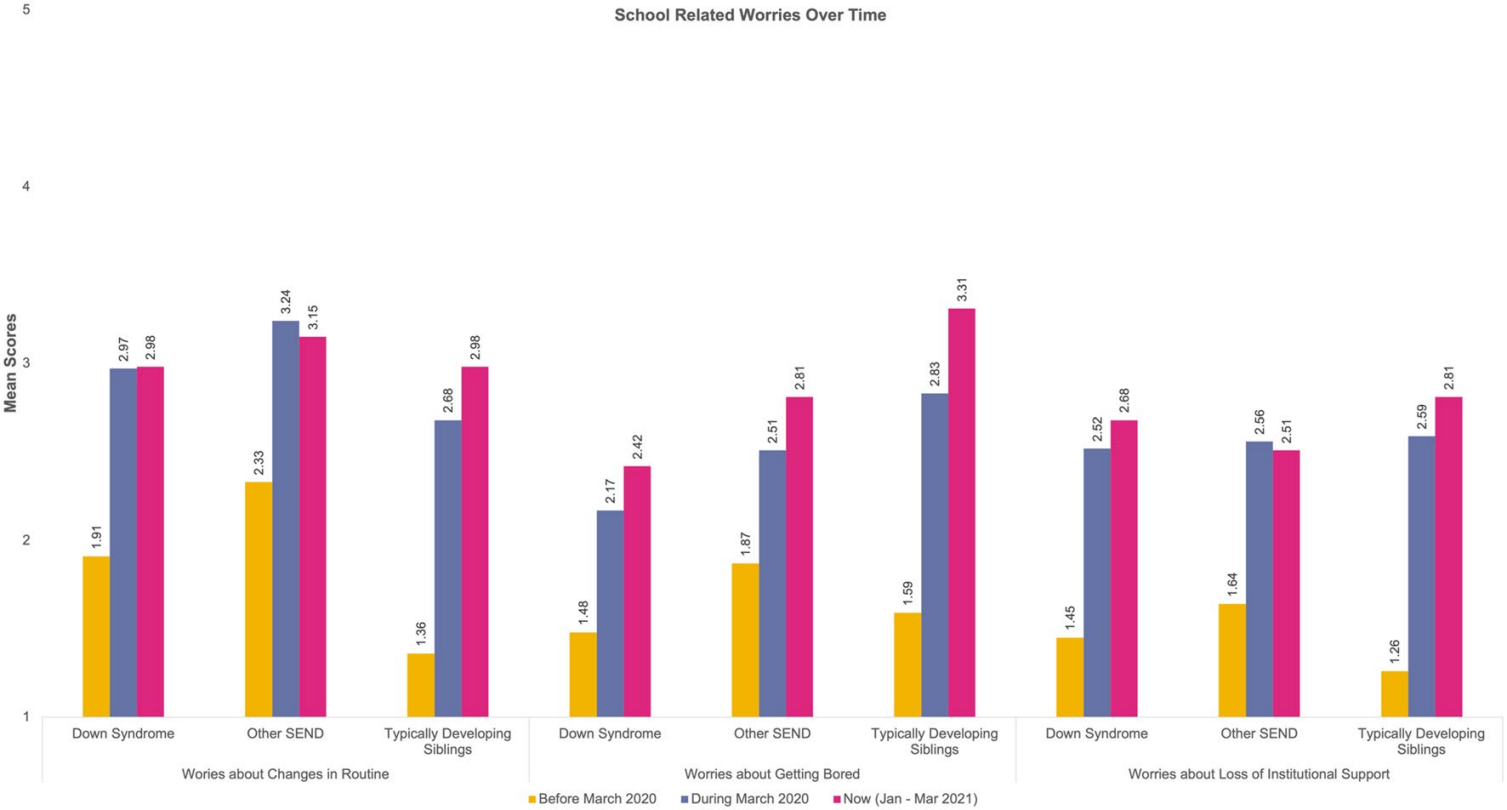
Visualisation of change over time for reported school related worries

**Fig. 5:**
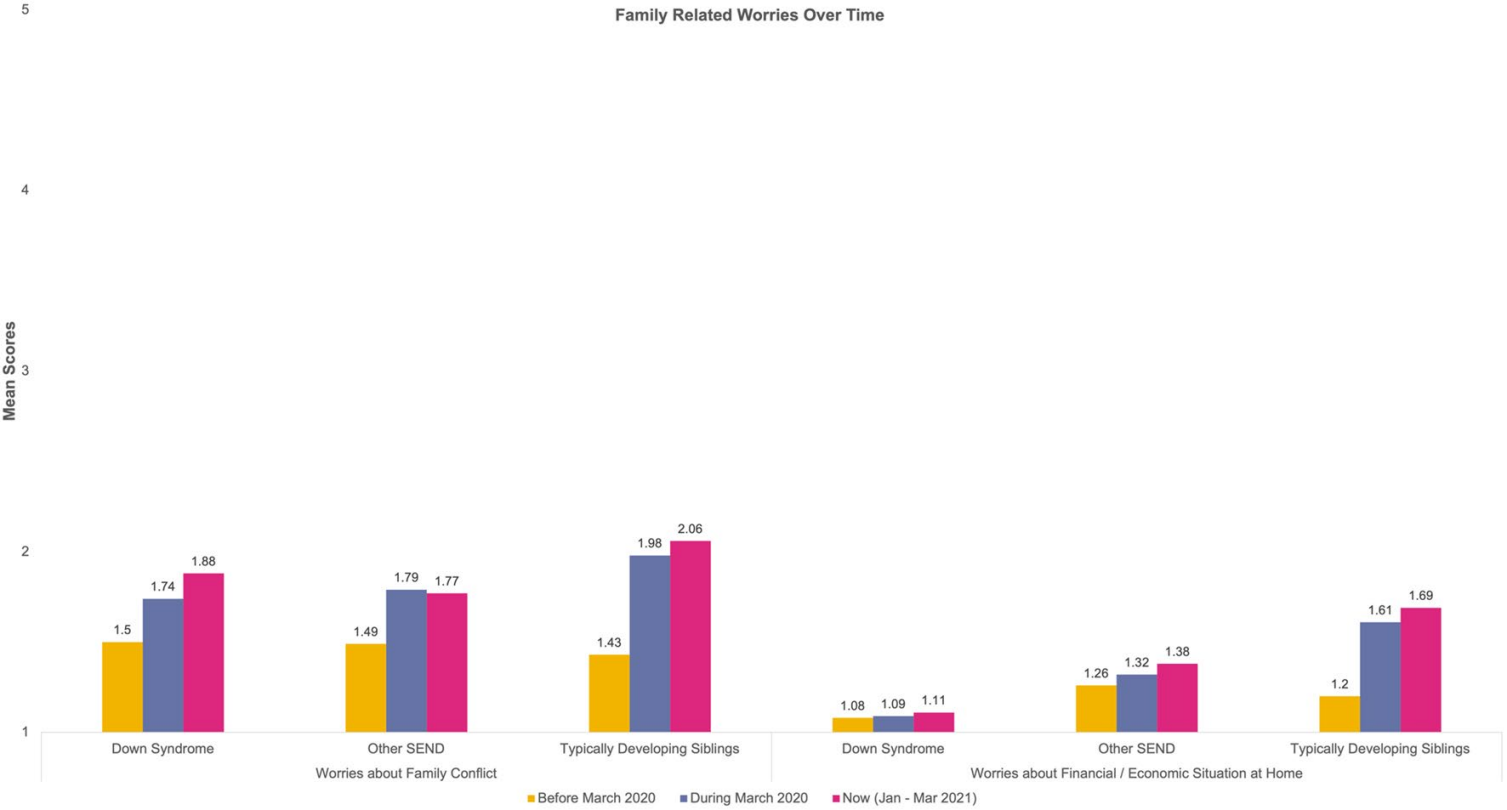
Visualisation of change over time for reported family related worries

Thirteen repeated measures 3 (Time) × 3 (Group) analyses were computed for the reported worries. Table 5 provides a detailed overview of the mean scores for each category in the worries. Sphericity violations and ANOVA outputs can be found on Table S1 in the supplementary materials. The change over time for our groups for each type of worry is presented in Fig. 2, 3, 4 and 5.

**Table 5:** Mean Scores and Standard Deviations of the Worries across the three time points and for the three groups (DS = Down Syndrome, TDS = Typically Developing Sibling., Other SEND)

	Time-point	Before March 2020			During March 2020			Now (Jan–Mar 2021)		
	Group	DS	Other SEND	TDS	DS	Other SEND	TDS	DS	Other SEND	TDS
Type of worries										
Health-related worries	Worries about illness in general	1.46 (.84)	2.15 (1.37)	1.52 (.88)	1.82 (1.28)	2.36 (1.42)	2.46 (1.38)	1.97 (1.37)	2.47 (1.50)	2.41 (1.32)
	Worries about COVID-19	1.23 (.65)	1.45 (1.06)	1.41 (.77)	2.12 (1.46)	2.45 (1.47)	3.09 (1.34)	2.30 (1.47)	2.45 (1.36)	2.96 (1.16)
	Worries about family’s safety with respect to COVID-19	1.29 (.80)	1.43 (,93)	1.57 (.96)	1.89 (1.39)	2.17 (1.43)	3.22 (1.50)	2.02 (1.39)	2.26 (1.44)	3.19 (1.48)
	Worries about their own health	1.44 (.91)	1.66 (1.05)	1.41 (.88)	1.70 (1.14)	2.02 (1.28)	2.28 (1.28)	1.91 (1.24)	2.11 (1.36)	2.24 (1.26)
	Worries about getting ill	1.35 (.84)	1.64 (1.13)	1.24 (.55)	1.94 (1.39)	2.09 (1.36)	2.43 (1.34)	2.08 (1.44)	2.19 (1.39)	2.30 (1.33)
	Worries about others getting ill	1.45 (1.01)	1.74 (1.13)	1.66 (1.02)	1.98 (1.51)	2.21 (1.49)	3.06 (1.50)	2.00 (1.48)	2.28 (1.51)	3.11 (1.48)
Social related worries	Worries about friends	1.74 (1.09)	1.83 (1.23)	1.80 (1.26)	2.94 (1.48)	3.13 (1.47)	3.36 (1.32)	3.45 (1.48)	3.33 (1.49)	3.89 (1.25)
	Worries about approach	1.50 (.96)	1.70 (1.19)	1.40 (.95)	2.65 (1.48)	2.63 (1.40)	3.09 (1.42)	2.98 (1.65)	2.80 (1.44)	3.19 (1.48)
Worries related to school closures	Worries about changes in routine	1.91 (1.25)	2.33 (1.43)	1.36 (.71)	2.97 (1.57)	3.24 (1.58)	2.68 (1.30)	2.98 (1.55)	3.15 (1.49)	2.98 (1.25)
	Worries about getting bored	1.48 (.85)	1.87 (1.19)	1.59 (1.06)	2.17 (1.33)	2.51 (1.52)	2.83 (1.28)	2.42 (1.49)	2.81 (1.57)	3.31 (1.31)
	Worries about loss of institution (including closure of school)	1.45 (.95)	1.64 (.125)	1.26 (.59)	2.52 (1.68)	2.56 (1.53)	2.59 (1.49)	2.68 (1.67)	2.51 (1.56)	2.81 (1.57)
Family-related worries	Worries about family conflict	1.50 (1.03)	1.49 (1.04)	1.43 (.72)	1.74 (1.13)	1.79 (1.18)	1.98 (1.25)	1.88 (1.27)	1.77 (1.07)	2.06 (1.37)
	Worries about finance/economic situation at home	1.08 (.27)	1.26 (.87)	1.20 (.45)	1.09 (.34)	1.32 (1.02)	1.61 (1.09)	1.11 (.40)	1.38 (1.15)	1.69 (1.15)

*p < .05, **p < .01, ***p < .001

#### Health-Related Worries

We measured multiple health-related worries. For the *Worries about Illness in General,* there was a significant increase over time (*p* < 0.001) and across the three groups (*p* < 0.05) as well as a significant Group x Time interaction (*p* < *0.0*01). A similar pattern, in terms of significance, was noticed for the *Worries about COVID-19; Worries about Others Getting Ill* and *Worries about Family’s Safety with Respect to COVID-19.* As can be seen in Fig. 2, the individuals with DS scored lower or similarly to their TDS on the previously mentioned worries. In addition, the repeated measures analysis revealed a significant Group × Time interaction for the *Worries about COVID-19* (*p* < *0.0*01*); Worries about Others Getting Ill* (*p* < 0.001) and *Worries about Family’s Safety with Respect to COVID-19* (*p* < 0.001).

On the *Worries about their Own Health*, there is a significant increase over time (*p* < 0.001) and a significant Group × Time interaction (*p* < 0.008), but there is not a significant difference between the groups. The difference between the groups is presented in Means/SD on Table 5 and is also visualised in Fig. 2. The repeated measures and mean scores show a similar pattern for the *Worries about Getting Ill;* a significant increase over time (*p* < 0.001) as well as a significant Group x Time interaction (*p* < 0.05).

#### Social-Related Worries

For *Worries about Friends,* there was a significant effect of time (*p* < 0.001) with individuals of DS worrying more about their friends’ interaction during time-point 3 compared to individuals with other SEND. Yet, both groups score lower than their TDS.

When looking at the *Worries about Approach,* we see the exact same pattern of worrying (time effect significant: *p* < *0.0*01). Individuals with DS worrying more about approach techniques during time-point 3 compared to the individuals with Other SEND, yet both groups scored lower than their TDS.

#### School-Related Worries

We also measured worries relating to schools. A significant effect of time was detected for both the *Worries about Changes in Routine* (*p* < 0.001), *Worries about Loss of Institutional Support* (*p* < 0.001), and *Worries about Getting Bored (p* < 001). This explains the different scores across time.

For the *Worries about Getting Bored,* there is also a significant effect of Group (*p* < 0.05*)*. In addition, there is also a significant Group * Time interaction for the *Worries about Changes in Routine (p* < 0.036) and the *Worries about Getting Bored (p* < *0.0*02). The individuals with DS scored significantly lower when compared to their TDS and the Other SEND groups; providing evidence that individuals with DS worry less. When considering TDS, they worried more about getting bored than their SEND siblings overall and then the other SEND group scored higher than the individuals with DS.

#### Family-Related Worries

*Worries about Family Conflict* were reported to change over time for all the individuals (*p* < 0.001), but there was no significant difference amongst groups nor an interaction between group and time. When looking at the *Worries about Financial / Economic Situation at Home*, not only there was a difference over Time (*p* < 0.001) but there was a significant Group effect (*p* < 0.01) and Group * Time interaction (*p* < 0.01). We can see differences amongst the groups for this worry over time but also between the groups. Specifically, individuals with DS scored lower when compared to the other two groups, whilst TDS scored higher across all three time-points compared to the other SEND group too.

## Discussion

To our knowledge this was the first study to explicitly focus on families of individuals with DS and examine the impact of the COVID-19 pandemic on caregivers’ reported levels of anxiety as well as anxiety and worries of individuals with DS compared to other SEND populations and their TDS. We also examined which factors could predict anxiety in all three groups (individuals with DS, other SEND and TDS) and the types of worries the three groups experienced during the COVID-19 pandemic. Taken together, these analyses improve our understanding on the impact of stressful life events (i.e., COVID-19 pandemic) and provide insight about the mental health of individuals with DS when compared to other SEND populations and TDS.

### Anxiety During COVID-19

When accounting for anxiety, our first hypothesis was verified, as individuals with DS scored lower for anxiety across all three time points than individuals with other SEND diagnoses. Such findings are in line with the theory that individuals with DS experience lower mental health adversities compared to other SEND populations (Gameren-Oosterom et al., 2011; Naerland et al., 2016). Furthermore, there was no significant difference between DS and TDS during the pandemic. Yet, the TDS group scored lower before the pandemic. However, in all three groups the same pattern was observed in that all three groups showed an increase in anxiety from before the pandemic to the start of the pandemic and these scores have so far not come down yet.

### Anxiety Predictors

For the DS group, health status, awareness of COVID-19 and an existing diagnosis of an anxiety disorder were strong predictors of anxiety during time-point 3. Despite the small number of those reporting an existing diagnosis of anxiety disorder in the DS group (n = 6), the magnitude of the relationship is strong enough to indicate its importance. Nonetheless, further examination is needed for individuals with DS with pre-existing anxiety disorders in order to obtain a fuller understanding of how the impact of COVID19 and their worries might differ.

Similarly, we noticed that for the other SEND group, the same factors predicted anxiety, although this time parental anxiety was also an important factor. Previous research has indeed shown that child related anxiety and parent anxiety do influence one another (Ashworth et al., 2019; Neece et al., 2012). However, our analyses show that caregivers’ anxiety is only one factor that explains raised anxiety in the other SEND group and that health status and awareness of COVID19 are factors that influence the anxiety of both groups. Whilst caregivers’ anxiety for the other SEND group was the lowest compared to the DS and TDS groups, it seemed to be a driving factor for anxiety in our other SEND model. This could be explained by the fact that indeed depending on the neurodevelopmental condition of the child, the caregiver experiences different levels of stress (Ashworth et al., 2019).

Only a pre-existing anxiety disorder was a strong factor of anxiety for the TDS group. However, some of the factors had to be omitted from our model due to the low number of reported cases (see the limitations discussed below). Nonetheless, our findings indicate that factors such as age and gender did not drive anxiety at all in any of our models.

As far as we can tell, this is the first study to provide evidence that COVID-19 awareness can predict anxiety in individuals with DS (see limitation and future studies section) which indicates that the more aware an individual with DS is of a stressful event (e.g., COVID-19 pandemic), the higher the levels of anxiety they will experience. This is in line with Sideropoulos et al. (2021) which showed that COVID-19 awareness, anxiety disorders and caregivers’ anxiety are associated with increased anxiety in other SEND populations. These results support claims made in previous studies that increased caregivers’ anxiety is linked to perceptions of children’s stress and anxiety symptoms (Platt et al., 2016; Russell et al., 2020). Nevertheless, there are other factors that could impact on caregivers’ perceptions of children’s stress. For instance, our data show that caregivers of individuals with DS had higher anxiety levels, yet caregivers reported lower anxiety for their DS children compared to the caregivers with children diagnosed with other SEND. This contradicts the literature on the Down Syndrome Advantage (Esbensen & Seltzer, 2011; Kasari & Sigman, 1997) and shows that the relationship between the caregivers’ anxiety/stress and the perception of their children’s anxiety/stress is multifaceted. A possible explanation of the high reported levels of anxiety in the caregivers with DS children group could be due to the lack of access to social network which, from previous research, seems to work as a preventing factor (Hauser-Cram et al., 2001).

### Worries

For our 3rd hypothesis, we expected to see individuals with DS to score lower on the worries compared to individuals with other SEND. However, our analyses showed a more complex picture and indeed individuals with DS scored differently on some worries but not all. For the health-related worries, we see a change over time for all groups (DS, other SEND and TDS) with individuals with DS scoring lower or similarly to the TDS group on COVID-19 related worries about their own or others’ health compared to the other SEND group. Worries around health have been found to be increased in SEND families and our study seems to provide further support to such claims (Asbury et al., 2021). As highlighted by Emes et al., (2021), individuals with DS are at greater risk for severe outcomes of COVID-19, nonetheless our data suggest that this is not a major concern for families and individuals with DS.

In terms of social-related worries, both individuals with DS and other SEND diagnoses worried more than the TDS group. Nonetheless, individuals with DS seem to exhibit greater worry about approaching others compared to the other SEND group and the TDS group. This might be linked to the well reported fact that individuals with DS are highly sociable and have good ‘people’ skills and that they look towards others when being faced with difficult and challenging tasks (see review by Cebula & Wishart, 2008). Seeing the loss of support during the COVID-19 pandemic, we could explain the elevated worries of approach for the DS group. Further evidence in the literature also highlights the importance of approach for individuals with DS and other SEND in general and how the lack of social interaction can lead to feelings of isolation (Houtrow et al., 2020), being overwhelmed due to loss of access to support workers who communicate better with individuals with SEND (Asbury et al., 2021), and other stressors that SEND families face. Specifically, for individuals with DS as they are experiencing a lot of sensory processing difficulties (Will et al., 2019; Barisnikov & Lejeune, 2018).

When looking at the school-related worries, all groups were revealed to be equally worried. However, individuals with DS worried less about boredom than the TDS and the other SEND groups. This suggests, although indirectly, that schools provide structured activities that protect both TDS and SEND groups from becoming bored (Jeste et al., 2020; Sideropoulos et al., 2021). However, individuals with DS reported similar worries in terms of loss of institutional support or changes in routines. This could be explained by the fact that many individuals with SEND (inclusive of DS) with health needs had to be shielded (Van Herwegen et al., 2020a, 2020b) and thus, experienced a loss of support that schools would normally provide.

Finally, for the family-related worries, the three groups reported similar worries with the exception of *Financial Situations* for which individuals with DS scored lower than the other two groups, while the TDS scored higher than the individuals with DS or other SEND diagnoses. It is evident from the WHO (2021) report that financial instability is an adversity that could lead to more stress and anxiety to the general population and that could explain the higher scores of TDS compared to the individuals with DS and other SEND, who tend to be less aware of financial situations and who struggle with mathematical concepts (Cuskelly et al., 2017; Faragher, 2017).

Overall, it was observed that individuals with other SEND diagnosis reported higher levels of worries compared to individuals with DS, replicating previous findings (Sideropoulos et al., 2021) as well as providing further evidence that individuals with DS seem to be less anxious and experience lower levels of worries compared to other SEND groups who have been shown to express increased worries in previous research (e.g., autism: Miniarikova et al., 2021). However, also for individuals with DS, anxiety and some areas of worries have increased as a result of the ongoing pandemic. The current study has shown that especially those who are aware of COVID19, have underlying health issues, and have existing anxiety disorders are at greater risk of showing higher anxiety.

## Impact and Conclusion

These findings highlight that not all SEND groups are equally affected in terms of anxiety and that minor differences in predictive factors as well as worries exist. This matters in terms of toolkits to be developed to support individuals with SEND during stressful times such as a health pandemic. However, despite the individuals with DS being less anxious at the beginning of the pandemic, they still have significant worries about certain aspects. Therefore, individuals with DS still need to be supported and have serious concerns that need to be addressed.

## Limitations and Future Studies

The present study investigated the anxiety levels and worries through caregiver report, rather than a self-reported measure. Our previous work, but also other research, shows the direct link between caregivers' anxiety and the children’s perception of mental health state (Sideropoulos et al., 2021). However, future studies should also examine the experiences of those with SEND diagnoses, including those with DS directly through self-reports.

Furthermore, only 6 participants with DS reported an existing diagnosis of anxiety compared to the other SEND participants who reported 19 cases, whilst this is a very small number for a factor to be used in a regression model, this could be indicative of what we might see in larger sample sizes for the DS population.

In addition, anxiety was measured using a non-standardised set of questions. Therefore, it is important that follow-up works use a standardised self-reported method to assess anxiety level of this population such as the Generalised Anxiety Disorder scale (Spitzer et al., 2006).

Finally, it is evident from our data that reported anxiety levels in all groups seem to plateau rather than decrease which is of great public health concern for the SEND community. However, this could be due to biased recall of the past. Another explanation could be that people who experienced higher/lower levels of anxiety did not participate in our online survey. Hence, future studies need to focus on (a) longitudinal designs and (b) the understanding of mental health of this population as well as on the prevalence factors and recovery from the pandemic’s impact. 

## Supplementary Information

Below is the link to the electronic supplementary material.

Supplementary file1 (DOCX 20 kb)
