# Supplementary material for: Anxiety and Worries of Individuals with Down Syndrome During the COVID-19 Pandemic: A Comparative Study in the UK
Source: J Autism Dev Disord. 2022 Feb 1;53(5):2021–36. doi: 10.1007/s10803-022-05450-0 (PMC8806133; doi:10.1007/s10803-022-05450-0)
Supplement: Supplementary file 1 — Supplementary file1 (DOCX 20 kb) [file 10803_2022_5450_MOESM1_ESM.docx]

Table 1S. Sphericity violations and ANOVA output for Worries

|  |  |  |  |  |  |  |  |  | | |
| --- | --- | --- | --- | --- | --- | --- | --- | --- | --- | --- |
| **Type of concern** |  | **Test of**  **Sphericity Checks** | **Adjustment Method and ε level** | **Source** | **d1** | **d2** | **F** | | **p** | **η^2^_p_** |
|  |  |  |  |  |  |  |  | |  |  |
| **Health-related worries** | Worries about COVID-19 |  |  |  |  |  |  | |  |  |
|  |  | χ^2^ (2) = 39.28 *p* < .001 | Huynh-Feldt correction (ε=.84) | Group | 38.12 | 164 | 5.4 | | <.006* | .061 |
|  |  |  |  | Time | 1.68 | 275.86 | 107.22 | | < .001*** | 0.40 |
|  |  |  |  | Group x Time | 3.36 | 275.86 | 3.78 | | <.008* | .044 |
|  | Worries about others getting ill | χ^2^ (2) = 56.12, *p* <.001 | Huynh-Feldt correction (ε=.79) | Group | 2.00 | 163 | 6.44 | | <.002* | 0.07 |
|  |  |  |  | Time | 1.58 | 257.25 | 63.45 | | <.001*** | 0.28 |
|  |  |  |  | Group x Time | 3.16 | 257.25 | 8.51 | | <.001*** | .095 |
|  | Worries about illness in general | χ^2^ (2) = 29.30, *p* <.001 | Huynh-Feldt correction (ε=.88) | Group | 2.00 | 165 | 3.71 | | <.027* | 0.04 |
|  |  |  |  | Time | 1.76 | 289.77 | 38.43 | | <.001*** | 0.19 |
|  |  |  |  | Group x Time | 3.51 | 289.77 | 5.16 | | <.001*** | 0.06 |
|  | Worries about their own health | χ^2^ (2) = 23.03, *p* <.001 | Huynh-Feldt correction (ε=.90) | Group | 2.00 | 164 | 1.43 | | <.243 | 0.02 |
|  |  |  |  | Time | 1.81 | 296.31 | 37.68 | | <.001*** | 0.19 |
|  |  |  |  | Group x Time | 3.61 | 296.31 | 3.70 | | <.008*** | 0.04 |
|  | Worries about family’s safety with respect to COVID-19 | χ^2^ (2) = 54.53, *p* <.001 | Huynh-Feldt correction (ε=.79) | Group | 2.00 | 164 | 11.48 | | <.001*** | 0.12 |
|  |  |  |  | Time | 1.59 | 260.53 | 82.66 | | <.001*** | 0.34 |
|  |  |  |  | Group x Time | 3.18 | 260.53 | 7.49 | | <.001*** | 0.08 |
|  | Worries about getting ill | χ^2^ (2) = 50.29, *p* <.001 | Huynh-Feldt correction (ε=.81) | Group | 2.00 | 163 | 0.61 | | .543 | 0.01 |
|  |  |  |  | Time | 1.61 | 262.61 | 61.81 | | <.001*** | 0.28 |
|  |  |  |  | Group x Time | 3.22 | 262.61 | 4.18 | | <.005** | 0.05 |
| **Social-related worries** | Worries about friends | χ^2^ (2) = 23.29, *p* <.001 | Huynh-Feldt correction (ε=.90) | Group | 2.00 | 162 | 2.10 | | .126 | 0.03 |
|  |  |  |  | Time | 1.80 | 292.01 | 164.43 | | <.001*** | 0.50 |
|  |  |  |  | Group x Time | 3.61 | 292.01 | 2.42 | | .055 | 0.03 |
|  | Worries about approach | χ^2^ (2) = 81.40, *p* <.001 | Huynh-Feldt correction (ε=.73) | Group | 2.00 | 162 | 0.44 | | .644 | 0.01 |
|  |  |  |  | Time | 1.46 | 236.33 | 138.04 | | <.001*** | 0.46 |
|  |  |  |  | Group x Time | 2.92 | 236.33 | 3.30 | | <.022* | 0.04 |
| **School-related worries** | Worries about loss of routine | χ^2^ (2) = 21.49, *p* <.001 | Huynh-Feldt correction (ε=.91) | Group | 2.00 | 11.86 | 2.90 | | 0.058 | 0.04 |
|  |  |  |  | Time | 1.82 | 292.61 | 88.97 | | <.001*** | 0.36 |
|  |  |  |  | Group x Time | 3.64 | 292.61 | 2.70 | | <.036** | 0.03 |
|  | Worries about loss of institutional support | χ^2^ (2) = 61.73, *p* <.001 | Huynh-Feldt correction (ε=.77) | Group | 2.00 | 162 | 0.00 | | .966 | 0.00 |
|  |  |  |  | Time | 1.55 | 250.64 | 91.71 | | <.001*** | 0.36 |
|  |  |  |  | Group x Time | 3.09 | 250.64 | 1.92 | | .126 | 0.02 |
|  | Worries about getting bored | χ^2^ (2) = 42.06, *p* <.001 | Huynh-Feldt correction (ε=.83) | Group | 2.00 | 164 | 3.83 | | <.024** | 0.05 |
|  |  |  |  | Time | 1.66 | 272.83 | 98.37 | | <.001*** | 0.38 |
|  |  |  |  | Group x Time | 3.33 | 272.83 | 4.77 | | <.002*** | 0.06 |
| **Family-related worries** | Worries about family conflict | χ^2^ (2) = 42.90, *p* <.001 | Huynh-Feldt correction (ε=.83) | Group | 2.00 | 164 | 0.27 | | .764 | 0.00 |
|  |  |  |  | Time | 1.66 | 271.93 | 30.65 | | <.001*** | 0.16 |
|  |  |  |  | Group x Time | 3.32 | 271.93 | 2.12 | | <.091 | 0.03 |
|  | Worries about financial / economic situation at home | χ^2^ (2) = 81.40, *p* <.001 | Huynh-Feldt correction (ε=.73) | Group | 2.00 | 163 | 4.97 | | <.008*** | 0.06 |
|  |  |  |  | Time | 1.53 | 249.82 | 10.04 | | <.001*** | 0.06 |
|  |  |  |  | Group x Time | 3.07 | 249.82 | 4.80 | | <.003*** | 0.06 |
|  |  |  |  |  |  |  |  |  |  |  |

| *Note.*  * p < .05, ** p < .01, *** p < .001 |
| --- |
|  |
